# Fragile X-Related Protein 1 (FXR1) Promotes Trophoblast Migration at Early Pregnancy via Downregulation of GDF-15 Expression

**DOI:** 10.1007/s43032-021-00693-1

**Published:** 2021-07-21

**Authors:** Wei Hong, Jin-Hong Chen, Hong-jiao Ma, Li-Li, Xiao-Cui Li

**Affiliations:** Department of Obstetrics and Gynaecology, Shanghai First Maternity and Infant Hospital, School of Medicine, Tongji University, Shanghai, 200092 China

**Keywords:** FXR1, GDF-15, Early pregnancy, Recurrent spontaneous abortion, Trophoblast migration

## Abstract

Fragile X-related protein 1 (FXR1) is an RNA-binding protein that can regulate specific mRNA decay in cells. Our previous study showed that FXR1 expression was significantly decreased in trophoblasts from patients with unexplained recurrent spontaneous abortion (RSA); however, the role of FXR1 in trophoblast function during early placenta development has not been fully elucidated. In this study, we found that knockdown of FXR1 using siRNA effectively inhibited the migration of HTR-8 cells and extravillous trophoblast (EVT) outgrowth in an ex vivo extravillous explant culture model. Furthermore, through analysis of a panel of cytokines, we found that the GDF-15 protein was upregulated after knockdown of FXR1 in HTR-8/SVneo cells. This was further confirmed by western blotting and immunofluorescence in HTR-8/SVneo cells and an extravillous explant. Our data also showed that FXR1 expression was downregulated and GDF-15 was upregulated in chorionic villous tissues from RSA patients compared with those from healthy controls (HCs). Further, immunohistochemistry showed a strong expression of GDF-15 in chorionic villous tissue in the RSA group, which was mainly distributed in villous trophoblasts (CTBs) and syncytiotrophoblasts (STBs). Moreover, knockdown of GDF-15 enhanced the migration of HTR-8 cells, while overexpression of GDF-15 using plasmid or treatment with recombinant human GDF-15 protein inhibited trophoblast migration. Importantly, RNA-binding protein immunoprecipitation showed that FXR1 directly bound to the 3′-UTR of *GDF-15* mRNA to promote *GDF-15* mRNA decay. Together, our data provide new insight into the function of FXR1 in human placenta via regulation of GDF-15 expression in trophoblasts and suggest a possible pathological process involved in RSA.

## Introduction

Implantation of the embryo is a key step to maintaining a successful pregnancy and depends on embryo quality; embryo hatching; trophoblast differentiation, migration, and invasion; uterine receptivity and proper maternal–fetal cross talk; and immune regulation [1–4]. Two major trophoblast cell lineages are identified as participating in the early stages of human placental development: villous trophoblasts (CTBs) and extravillous trophoblasts (EVTs). CTBs are trophoblast progenitor cells that can differentiate to syncytiotrophoblasts (STBs) or EVTs. Two EVT populations can be identified; interstitial cytotrophoblasts (iCTBs) invade the decidual stroma, whereas endovascular cytotrophoblasts (eCTB) colonize the maternal spiral arteries [1, 5–7]. Defective EVT migration and invasion can lead to abnormal placentation and abnormal maternal–fetal connection, which causes adverse pregnancy outcomes, such as first trimester recurrent spontaneous abortion (RSA), placental insufficiency, pre-eclampsia, and intrauterine growth restriction [1, 3–9]. RSA, defined as two or more unexplained recurrent spontaneous abortion before 20 weeks of gestation, affects 1–5% of couples and can cause both physical and emotional distress [5, 6]. Causes of RSA include anatomical factors and embryo chromosomal abnormalities, but the pathogenic factors of some unexplained recurrent miscarriages are still unknown.

Fragile X-related protein 1 (FXR1) is a homologue of the fragile X mental retardation syndrome protein (FMRP) and interacts with FXR2 to form the fragile X-related (FXR) family of RNA-binding proteins [10, 11]. FXR1 is associated with regulation of specific mRNAs in cells and is involved in the pathogenesis of various diseases. In our previous study, we found that *FXR1* mRNA expression level was significantly decreased in trophoblasts from RSA patients and that FXR1 is involved in the regulation of the maternal fetal interface by binding to the 3′-UTR region of COX-2 to repress its expression [12]. However, the role of FXR1 in early placenta development has not been fully elucidated.

Growth differentiation factor 15 (GDF-15) is a divergent member of the human transforming growth factor-β (TGF-β) superfamily, which is also known as MIC-1 and placental transforming growth factor beta (PTGFB) [13, 14]. The most abundant source of MIC-1 mRNA transcripts is the human placenta [15]. Intense MIC-1 immunostaining was observed in the STBs and the trophoblast cell columns that develop into the invasive EVTs of the first trimester placenta [15, 16]. GDF-15 is also present in stromal cells and luminal and glandular epithelial cells of the first trimester decidua [16, 17]. GDF-15 is suggested to help maintain pregnancy by suppressing the production of proinflammatory cytokines and regulating trophoblast invasion [15–19].

Together, in this study, we found that FXR1 affect trophoblast migration by regulating GDF-15 expression in trophoblasts. Further, we clarified the relationship between FXR1 and GDF-15 expression. These results suggest that dysregulation of FXR1 expression may be associated with the pathogenesis of RSA disease.

## Materials and Methods

### Patient Characteristics

Twelve patients, aged 28–36 years old (mean age, 31 ± 3.17 years), who had suffered RSA and were treated at the Department of Obstetrics and Gynaecology at Shanghai First Maternity and Infant Hospital at the Tongji University School of Medicine between May 2017 and February 2018 were included in this study. RSA patients with gestational age of 6–12 weeks were confirmed missed abortion by ultrasound. When inevitable abortions occurred, they were treated with dilatation and curettage of the uterus, and villous tissue samples were collected. Chromosomal microarray analysis of villi tissue was normal. Ten females aged 25–32 years old (mean age, 28.8 ± 2.57 years), with normal early pregnancies were recruited as healthy controls (HCs). These patients underwent artificial abortion to terminate their unwanted pregnancies at 6–12 weeks of gestation, and samples of villi tissues were collected. All patients in our study with following comorbidities have been excluded: embryo chromosomal abnormalities, anatomic, anatomical, and immune abnormalities, as well as complications such as hypertension, diabetes, thyroid abnormalities, infection with chlamydia, ureaplasma in cervical mucus, alcohol abuse, tabacco abuse, and obesity (BMI > 30). The samples were paraffin embedded or stored in liquid nitrogen until analysis or used immediately for further extravillous explant culture. The protocol of this study was approved by the Medical Ethics Committee at Shanghai First Maternity and Infant Hospital, Shanghai (ethics protocol number KS1665). Written informed consent was obtained from all of the participants before enrollment.

### Cell Culture

The HTR-8/SVneo cell line [20], which is derived from human invasive extravillous trophoblasts, was kindly gifted by Dr. P.K. Lala (University of Western Ontario, London, Ontario, Canada). Cells were cultured in DMEM /F12 containing 10% fetal bovine serum (FBS) and all tissue culture reagents were purchased from Gibco company.

### Knockdown of FXR1 and GDF-15

Knockdown of FXR1 was performed using FXR1 siRNA (sc-35423, Santa Cruz Biotechnology). The negative control used siRNA (sc-37007, Santa Cruz Biotechnology). The siRNAs were transfected into cells at a final concentration of 100 nmol/L using Lipofectamine RNAi_MAX_ transfection reagent (Invitrogen). GDF-15 knockdown was performed using a specific siRNA (siGDF-15) purchased from GenePharma, Inc (Shanghai, China) and was transfected into cells at a final concentration of 50 nmol/L. The FXR1 siRNA (h) sc-35423 is a pool of 3 different siRNA duplexes: sc-35423A: 5′-CUAGGAAUCUCGUUGGAAAtt-3′; sc-35423B: 5′-GGAAUGACUGAAUCUGAUAtt-3′; sc-35423C: 5′-GGAUCCUGAAGAAAUCAUAtt-3′. The GDF-15 siRNA sequence was as follows: (5′-CUCAGAGUUGCACUCCGAATTUUCGGAGUGCAACUCUGAGTT-3′).

### Overexpression of FXR1 and GDF-15

To generate the FXR1 overexpression construct, the coding sequence (CDS) of human FXR1 (NM_005087.4) was cloned into the pLvx-IRES-ZsGreen vector (Clontech Laboratories). To generate a construct overexpressing GDF-15, the CDS of human GDF-15(NM_004864.4) was cloned into the GV365 vector. We used the INTERFERin Transfection Reagent (Polyplus) to transfect pLvx-IRES-ZsGreen-FXR1, pCMV-GDF-15, and the control vector into cells.

### Cytokine Array Analysis

FXR1 was knocked down in HTR-8/SVneo cells using FXR1 siRNA. After 48 h, the cell culture supernatants were collected, and particulates were removed by centrifugation for further analysis using the human XL cytokine array kit (ARY022, R&D Systems) according to the recommended protocol.

### Microscope Imaging

HTR-8/SVneo cells were cultured on poly-L-lysine-coated coverslips in 24-well plates for 24 h and were transfected with control siRNA, FXR1 siRNA, control vector, or the FXR1 overexpression plasmid and cultured for an additional 48 h. The cells were washed three times with phosphate-buffered saline (PBS), fixed in 4% paraformaldehyde, washed three times with PBS, and stained with primary antibodies using standard immunofluorescence protocols. Images were captured and assessed using a Leica microscope. Primary antibodies for immunofluorescence included anti-FXR1 (Abcam) and anti-GDF-15 (NBP1-81050, Novus) at a 1:200 dilution.

### Quantitative Real-Time PCR (qRT-PCR)

We used TRIzol reagent (Life Technologies) to extract total RNA from cultured HTR-8/SVneo cells according to the manufacturer’s protocol. The total RNA, was treated with DNase, was reversed transcription using the PrimeScript™II first-strand cDNA synthesis kit (TAKARA) with oligo-dT primers and the cDNA was generated. QRT-PCR was performed using a SYBR green kit (Qiagen Inc.). Relative expression was calculated using the 2^−ΔΔCt^ method, and normalized to the internal control gene glyceraldehyde-3-phosphate dehydrogenase (*GAPDH*; human). The primers were as follows: FXR1 F: 5′-GAGAAGACGG-TATGGTTCCAT-3′; R: 5′-AGGCGTTCCATTCTTAGCTGT-3′. GAPDH F: 5′-CACTGG-GCTACACTGAGCAC-3′, R: 5′-AGTGGTCGTTGAGGGCAAT-3′

### Western Blot Analysis

Trophoblasts were washed twice with ice-cold PBS and proteins were isolated using 1% Triton X-100 reagent (Sigma-Aldrich; Merck, Germany) on ice. The lysate was centrifuged at 12,000 rpm at 4 °C for 25 min. The supernatant was obtained, and the protein concentration was detected using a BCA kit (Pierce, Rockford, IL, USA). Equal amounts of protein were subjected to 10% SDS polyacrylamide gel electrophoresis, followed by electrotransferring onto PVDF membranes (Pall Corporation, Ann Arbor, MI, USA). Then, 5% fat-free milk powder in TBS with 0.1% Tween-20 (Sigma-Aldrich; Merck, Germany) was used to block the PVDF membranes for 1 h. Antibodies FXR1 (diluted 1:1000; Abcam) and GDF-15 (diluted 1:1000; Abcam) were used to perform western blot analysis using standard techniques. Antibodies against β-tubulin and GAPDH (diluted 1:1000; Yeasen, Shanghai, China) were used as loading controls. ECL imaging system utilized was an Amersham Imager 600.

### RNA-Binding Protein Immunoprecipitation

RNA-binding protein immunoprecipitation (RIP) was conducted using the EZMagna RIP kit (Millipore, cat: 17-701), according to the manufacturer’s protocol. In brief, HTR-8/SVneo cells were lysed using RIP lysis buffer. RIPAb^+^ FXR1(Millipore) and nonspecific control rabbit IgG antibodies were used for the immunoprecipitations. RIP lysates and antibody bound to magnetic beads were incubated together overnight at 4 °C with rotation. Then, proteins in the immunoprecipitates were digested with proteinase K. Bound RNA was purified from the supernatant, reverse transcribed, and evaluated by qRT-PCR. Relative RNA expression was calculated using the 2^−ΔΔCt^ method and normalized against input values. The primers for GDF-15 are as follows: F: 5′-TCAGATGCTCCTGGTGTTGC-3′; R: 5′-GATCCCAGCCGCACTTCTG-3′.

### Immunohistochemistry

First trimester villous tissues were obtained from 12 RSA patients and ten HC group patients undergoing legal therapeutic abortion. Fragments of villous tissue were dissected free of embryonic and decidual tissues and washed three times with PBS. Subsequently, tissue samples were processed in routine paraffin embedding. Tissue section thickness was 5 μm. Slides were deparaffinized and rehydrated, boiled in EDTA buffer (pH 9.0) in a water bath at 95 °C for 20 min to retrieve antigen. Endogenous peroxidase activity was blocked with 0.3% hydrogen peroxide for 20 min. Slides were blocked with 5% FBS for 30 min and incubated with anti-GDF-15 (diluted 1:100, Abcam) at 4 °C overnight. After washing in PBS, the sections were incubated with biotinylated secondary antibody and stained by using a MaxvisionTM2 HRP-polymer anti-mouse/rabbit IHC kit (Fuzhou, China). Meyer’s hematoxylin (Sigma-Aldrich, St. Louis, MO) was used as a counterstain dye. An isotype controls (IgG) (ab172730, Abcam) had been used as negative control. Images were captured with the Leica microscope (Leica, Buffalo Grove, IL).

### Cell Migration

We evaluated the migration ability of trophoblasts using the transwell cell migration assay, according to standard protocols. Briefly, HTR-8/SVneo cells were transfected with siCtrl, siFXR1, siGDF-15, control vector, the FXR1 overexpression plasmid, or the GDF-15 overexpression plasmid and cultured for 24 h. To perform the transwell cell migration assay, 0.8 × 10^5^ cells in 200 μL of DMEM/F12 containing 1% FBS were placed into the upper chamber of each insert. The lower chambers were filled with 800 μL of DMEM/F12 containing 15% FBS, and the cells were incubated at 37 °C for 48 h. Then, the inserts were removed and washed in PBS three times. The inserts were fixed in 4% paraformaldehyde (Sigma-Aldrich) and stained with 0.1% crystal violet (Sigma-Aldrich). The non-migrating cells in the upper surface of the filter were removed by gently wiping with a cotton bud. The cells on the lower surface were observed using an inverted phase-contrast microscope (Leica). The number of cells that had invaded the lower surface were counted at a magnification of × 200. Each experiment was performed in duplicate, and the experiments were independently repeated three times.

### Wound Healing Assay

HTR-8/SVneo cells were transfected with siCtrl, control vector, or the FXR1 overexpression plasmid and plated in 6-well plates until they achieved about 90% confluence. A 1-mL pipette tip was uses to create scratch wounds, washed three times with PBS, and the culture medium was replaced with DMEM/F12 containing 1% FBS. For each image, wound gap distances between the two sides were measured randomly at six points using the ImageJ software calibrated for the image magnification. The average value was recorded and analyzed. The experiment was independently repeated three times. All experiments were independently repeated for three times.

### Extravillous Explant Culture

Extravillous explant culture is an ex vivo explant culture model created to evaluate trophoblast (especially EVT cells) migration and invasion [20–23]. Small 2–3 mm tissue sections were obtained from the tips of first-trimester human placental villi (6–10 weeks), dissected, and explanted in 24-well culture dishes pre-coated with phenol red-free Matrigel® substrate (Corning Life Sciences, New York, NY). Villi explants were cultured in DMEM/F12 media containing 10% FBS and 1% antibiotic–antimycotic (Gibco), successfully anchored on Matrigel matrix, and initiated outgrowths were used for subsequent experiments. EVT sprouting and migration from the distal end of the villous tips were observed and recorded daily. The extent of migration was measured via the ImageJ software. To test the effect of FXR1 on the migration of EVTs, 250 nM siRNA specifically targeting FXR1 or an equal concentration of control siRNA was introduced into two wells of extravillous explants from HC patients. The images were obtained after 24 h and 48 h of in vitro culture under a light microscope. The explant experiments with cultured villi were repeated three times.

### Statistical Analysis

All statistical values were calculated using SPSS 22.0 (Chicago, IL). Comparison between two groups was done using the independent sample *t* test. Normality of data was assessed with a one-sample Kolmogorov–Smirnov test. A *P* value of < 0.05 was considered statistically significant.

## Results

### FXR1 Regulated the Migration Ability of HTR-8 Cells

**Fig. 1:**
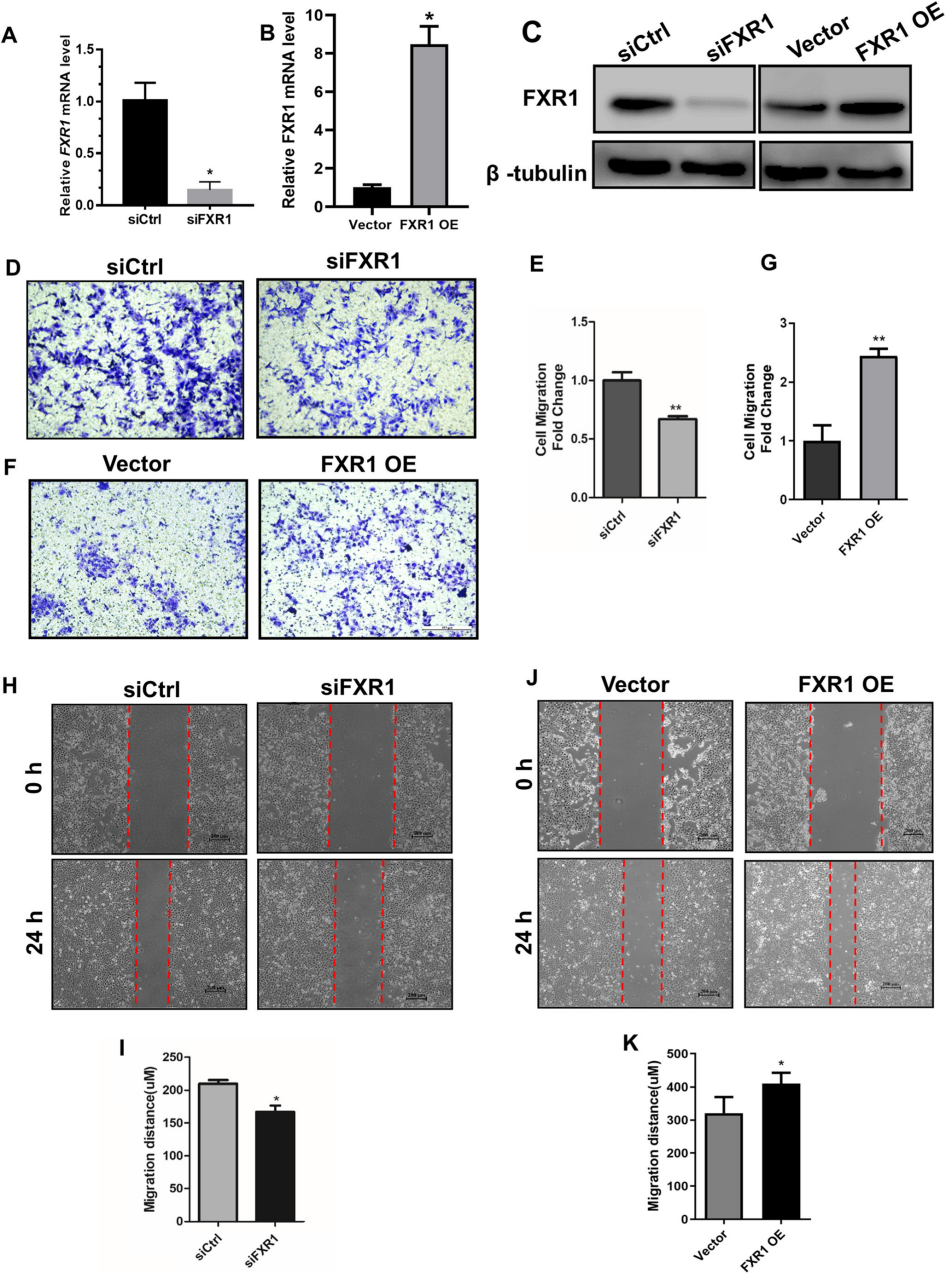
Knockdown of FXR1 inhibits the migration ability of HTR-8 cells. **A**, **B** QRT-PCR analysis of *FXR1* mRNA level in HTR-8/SVneo cells transfected with siCtrl, siFXR1, control vector, or FXR1-overexpressing vector after 48 h. **C** Western blotting analysis of FXR1 expression in HTR-8 cells transfected with siCtrl, siFXR1, control vector, or FXR1-overexpressing vector after 48 h. **D**, **E** Knockdown of FXR1 significantly reduced cell migration compared with the control cell line. **F**, **G** Overexpression of FXR1 in HTR-8/SVneo cells increased cell migration compared with that of the vector control cell line (bar = 100 μm). **H**, **I** FXR1 knockdown reduced the amount of wound closure in comparison with that of the control cell line. **J**, **K** Overexpression of FXR1 in HTR-8/SVneo cells resulted in increased wound closure ability compared with the vector (bar = 200 μm) ( *t* test, * *P* < 0.05, **P < 0.01)

To investigate whether FXR1 is involved in the migration of trophoblasts, the HTR-8/SVneo (HTR-8) cell line was transfected with siFXR or the FXR1 overexpression plasmid. Knockdown and overexpression of FXR1 was detected by western blotting and qRT-PCR. The control group siRNA and vector were transfected at the same time (Fig. 1A–C). Transwell cell migration and wound healing assays were also conducted. Knockdown of FXR1 significantly suppressed the migratory ability of HTR-8/SVneo cells (Fig. 1D–G), while FXR1 overexpression enhanced the migratory ability of HTR-8 cells (Fig. 1H–K).

### FXR1 Regulates Extravillous Trophoblast Outgrowth in an Ex Vivo Extravillous Explant Culture Model

**Fig. 2:**
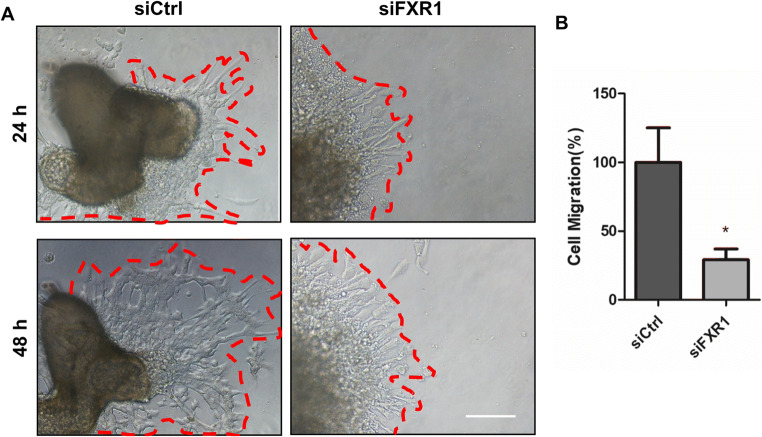
FXR1 regulates extravillous trophoblast outgrowth in an extravillous explant culture model. **A** Extravillous explants were obtained from HCs at 6–8 weeks of gestation and cultured on Matrigel. Serial pictures of the explants incubated with siFXR1 or siCtrl were taken under a light microscope after 24 and 48 h of culture (bar = 100 μm). **B** Statistical analysis of the migration distance of villous tips (%). Data are presented as mean ± SD of three independent experiments (*t* test, * *P* <0.05)

To further clarify the role of FXR1 in trophoblasts ex vivo, extravillous explants from first-trimester villi tissue samples (6–10 weeks of gestation) were cultured in Matrigel-coated 24-well dishes. Following 24 h of culture, the explants were anchored into the Matrigel and started to exhibit outgrowth. They were then transfected with control siRNA or siFXR1. At 48 h, the explants treated with siFXR1 outgrowths were significantly shorter than the siCtrl-treated explants (Fig. 2A, B). These results clearly suggest that FXR1 plays an important role in the regulation of trophoblast invasion in an ex vivo extravillous explant culture model.

### FXR1 Regulated GDF-15 Expression in HTR-8 Cells Posttranscriptionally

**Fig. 3:**
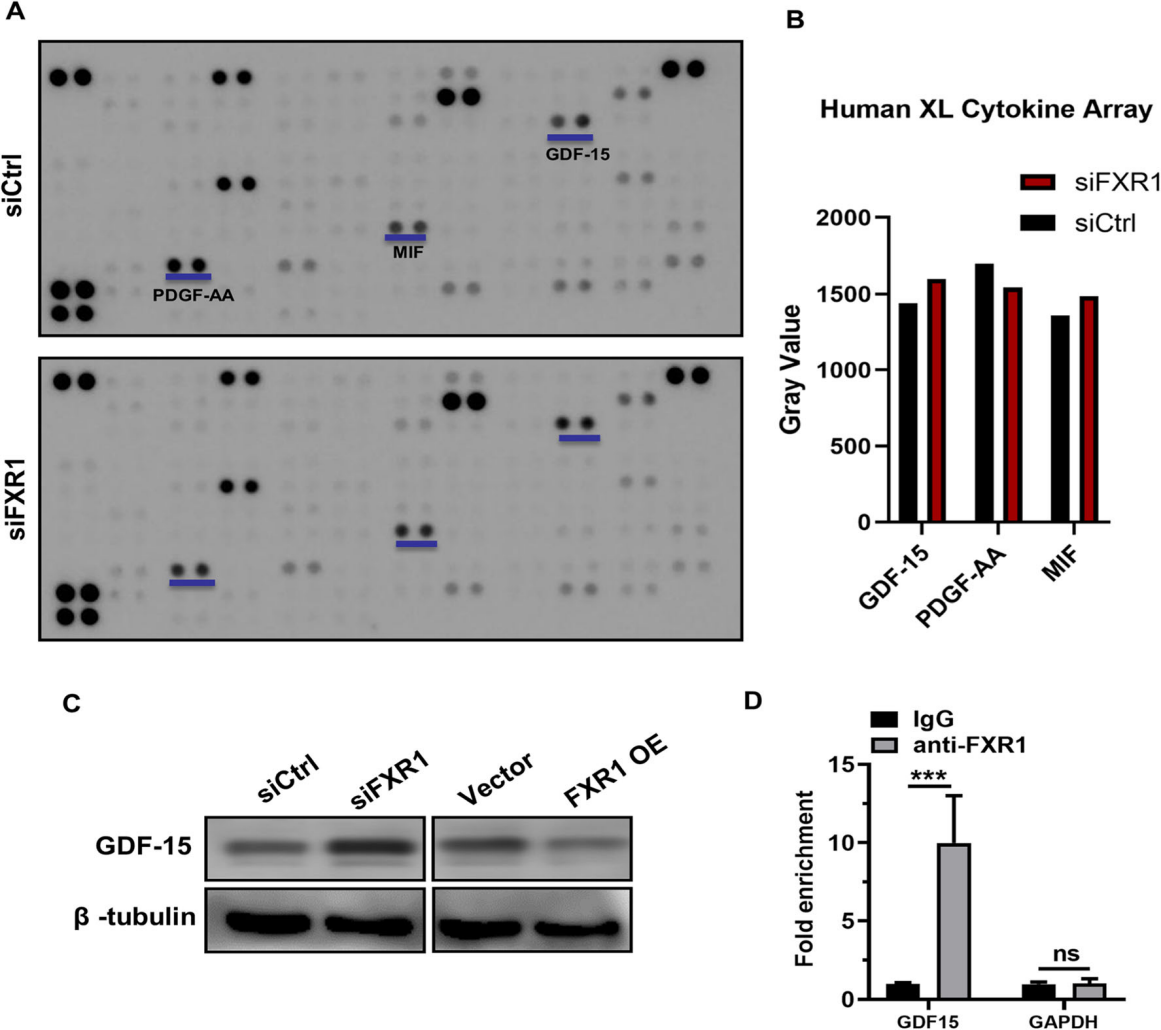
GDF-15 is regulated by FXR1 in HTR-8. **A**, **B** Human XL Cytokine Array Kit (ARY022 from R&D Systems) detected multiple cytokines in cell culture supernatants. The supernatants were obtained from HTR-8/SVneo cells that were transfected with siCtrl and siFXR1. The graph summarizes the relative signal intensity of the indicated molecules. Among them, GDF-15, PDGF-aa, and MIF varied obviously. **C** Western blotting analysis of GDF-15 expression in HTR-8/SVneo cells transfected with siCtrl, siFXR1, control vector, or FXR1-overexpressing vector after 48 h. **D** Analysis of co-purified RNA and respective enrichment as determined by qRT-PCR after anti-FXR1 or anti-IgG immunoprecipitation validated specific binding of *GDF-15* or *GAPDH* mRNA to FXR1. All immunoprecipitation experiments were done in biological replicates (*n* = 3). (*t* test, *P < 0.05)

To further explore the downstream genes regulated by FXR1 in trophoblasts, HTR-8 cells were transfected with siCtrl and siFXR1. After 48 h, we collected trophoblast culture supernatants, removed particulates by centrifugation, and screened them using a human cytokine array kit. The results showed that GDF-15 expression was obviously enhanced via calculating the gray value of the spots. Additionally, there were some visual differences in PDGF-aa and macrophage migration inhibitory factor (MIF) expression that need to be further verified and studied (Fig. 3A, B). To further confirm whether GDF-15 was regulated by FXR1, HTR-8/SVneo cells were transfected with FXR1 siRNA and an FXR1-overexpressing plasmid. After 48 h, the level of GDF-15 expression was detected by western blotting. These results showed that expression of GDF-15 was increased after knockdown of FXR1, while overexpression of FXR1 had the opposite effect (Fig. 3C). We further performed RNA immunoprecipitation assays to investigate the role of FXR1 in regulation of GDF-15 expression. FXR1 was transiently overexpressed in HTR-8/SVneo cells and immunoprecipitated with anti-FXR1. After isolation of the co-purifying RNA, enrichment of selected transcripts was measured by qRT-PCR, and we confirmed the specific enrichment of GDF-15 (Fig. 3D).

**Fig. 4:**
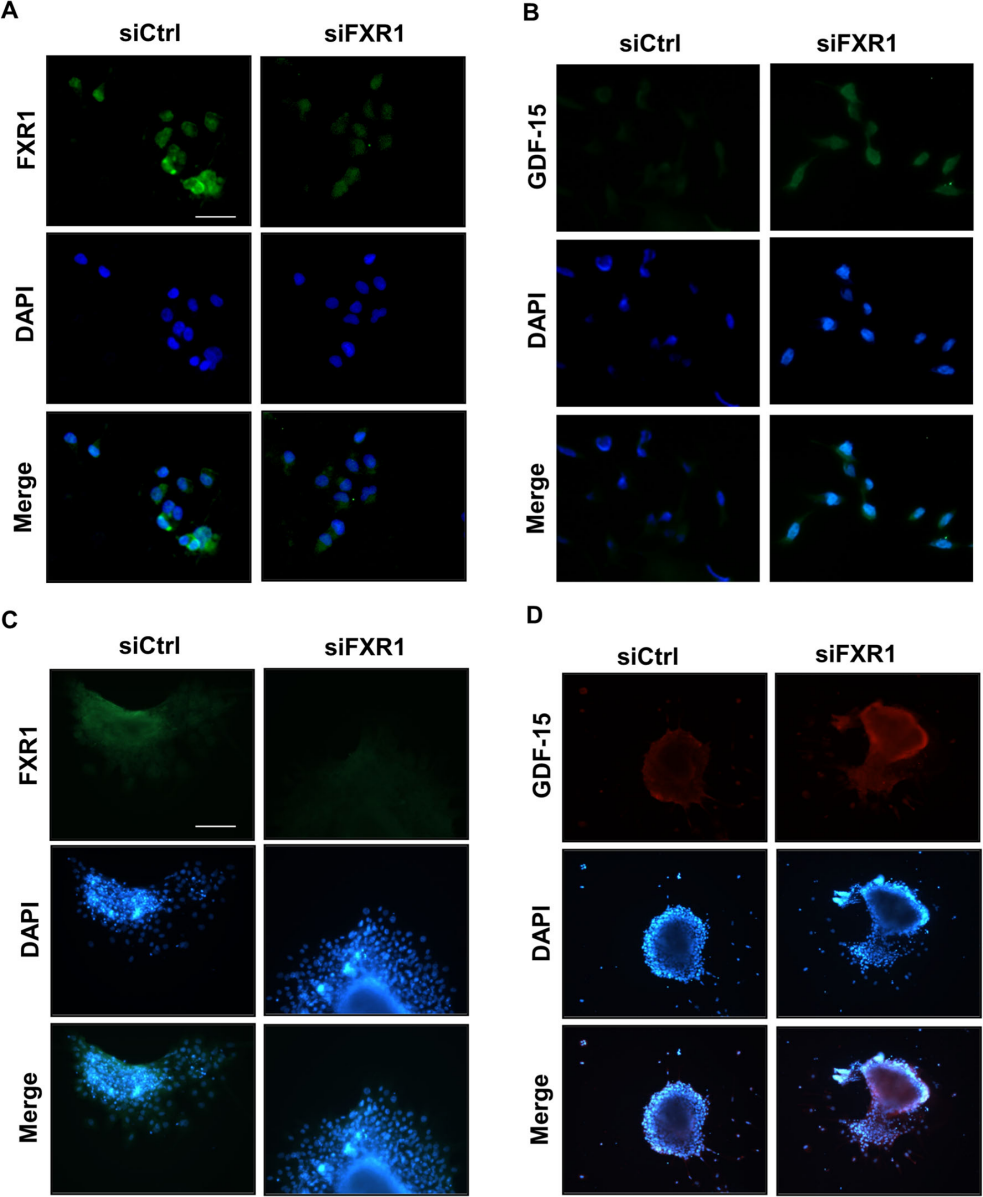
GDF-15 is regulated by FXR1 in trophoblasts. **A** Immunofluorescence staining using anti-FXR1 antibodies showed an obvious knockdown of FXR1 in HTR-8/SVneo cells transfected with siFXR1 compared to the siCtrl group. Green fluorescence signals indicate bound anti-FXR1 antibodies. DAPI-stained nuclei are blue. **B** Immunofluorescence staining using anti-GDF-15 antibodies showed an obvious increase in GDF-15 protein level in HTR-8/SVneo cells transfected with siFXR1 compared to the siCtrl group. Green fluorescence signals indicate bound anti-GDF-15 antibodies. DAPI-stained nuclei are blue. **C**, **D** Immunofluorescence staining showed an obvious knockdown of FXR1 and an obvious increase in GDF-15 protein level in extravillous explants cultured on Matrigel for 48 h incubated with siFXR1 compared to the siCtrl. Green fluorescence signals indicate bound anti-FXR1 antibodies. Red fluorescence signals indicate bound anti-GDF-15 antibodies. The DAPI-stained nuclei are blue. (**A**, **B** bar = 50 μm; **C**, **D** bar = 100 μm)

Immunofluorescence confirmed that GDF-15 is upregulated in HTR-8/SVneo cells after knockdown of FXR1 (Fig. 4A, B). We used extravillous explants instead of primary trophoblast extractions in vitro. In extravillous explants, placental villi that successfully anchored on the Matrigel matrix-initiated outgrowth, and EVT begin to sprout and migrate 24 h after explant. FXR1 siRNA and an equal concentration of control siRNA were introduced into four wells of extravillous explants from HC patients. After 48 h, immunofluorescence showed that GDF-15 was upregulated in extravillous trophoblasts after knockdown of FXR1 (Fig. 4C, D).

### FXR1 Expression Was Negatively Related with GDF-15 Expression in RSA Patients

**Fig. 5:**
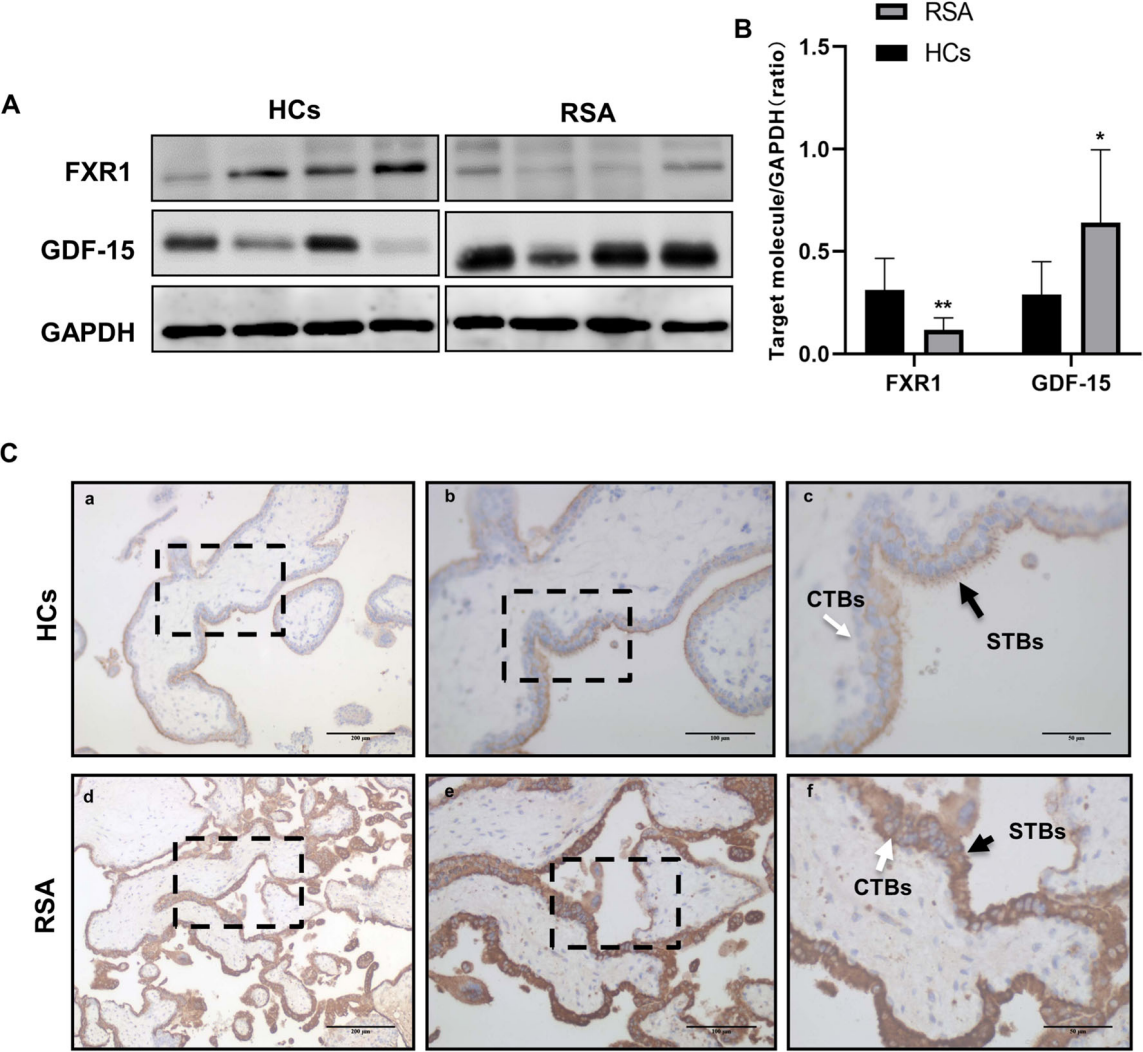
FXR1 is downregulated while GDF-15 is upregulated in RSA patients*.* **A**, **B** FXR1 and GDF-15 levels in first-trimester villous tissues from the RSA (*n* = 12) and HC groups (*n* = 10) were determined by western blotting (*t* test, *P < 0.05). Single staining of maternal villi (CTBs and STBs) using anti-GDF-15 antibody was visualized with the MaxvisionTM2 HRP-polymer anti-mouse/rabbit IHC kit. Sections were counterstained with hematoxylin. (a, d) Bar = 200 μm. (b, e) Bar = 100 μm. (c, f) Bar = 50 μm

In our previous study, immunohistochemical analysis of first-trimester chorionic villi tissues showed that FXR1 expression was significantly downregulated in RSA patients compared with that in HCs. At the mRNA level, FXR1 was downregulated in villi from the RSA group compared with those from HCs [12]. In this study, western blotting further confirmed that the level of FXR1 protein expression was downregulated in chorionic villous tissue from RSA patients compared to HCs, which is consistent with previous immunohistochemical and mRNA results. The level of GDF-15 protein was upregulated in chorionic villous tissue from RSA patients compared with HCs (Fig. 5A, B). Immunohistochemical analysis of paraffin-embedded first-trimester chorionic villous tissues was performed to further investigate the localization of GDF-15 protein. A strong expression of GDF-15 was observed in chorionic villous tissue from the RSA group, with the staining mainly distributed in CTBs and STBs (Fig. 5C). These results indicate that FXR1 and GDF-15 expression are important factors participating in the pathogenesis of RSA.

### Knockdown of FXR1 Inhibits Trophoblast Migration by Regulating GDF-15 Expression

**Fig. 6:**
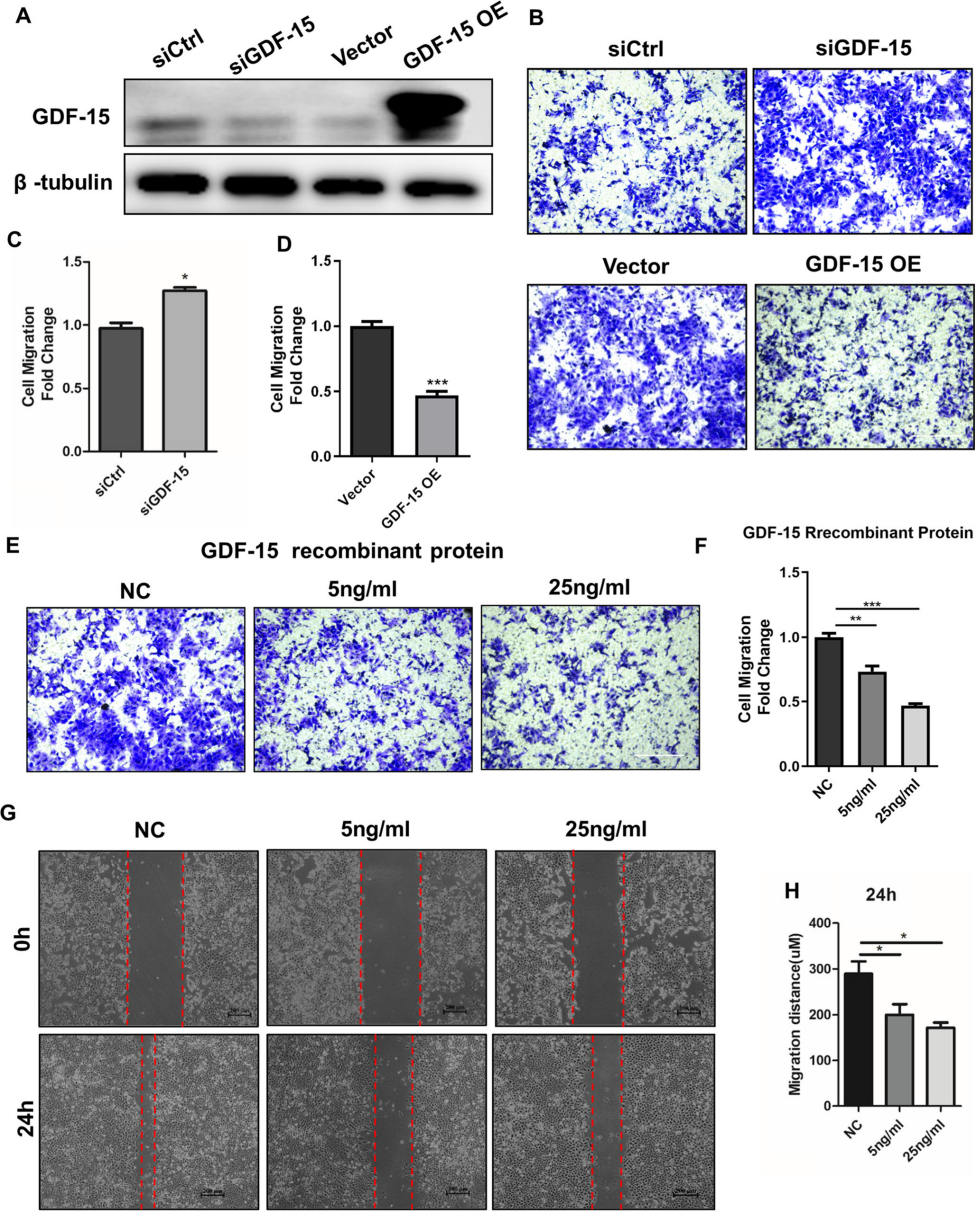
GDF-15 inhibit HTR-8 migration in trophoblasts. **A** Western blot analysis of GDF-15 expression in HTR-8/SVneo cells transfected with siCtrl, siGDF-15, control vector, or GDF-15-overexpressing vector after 48 h. **B**–**D** Knockdown of GDF-15 significantly reduced cell migration compared with the control cell line. Overexpression of GDF-15 increased cell migration compared with the vector control cell line (bar =100 μm). **E**, **F** The migration ability of HTR-8/SVneo cells was reduced by the addition of 5 ng/mL and 25 ng/mL GDF-15 recombinant protein (bar = 100 μm). **G**, **H** Adding GDF-15 recombinant protein reduced the extent of wound closure in the HTR-8/SVneo cell line

To further study role of GDF-15 in trophoblasts, HTR-8 cells were transfected with GDF-15 siRNA, siCtrl, a GDF-15-overexpressing plasmid, or a control vector. The knockdown and overexpression of GDF-15 at the protein level were confirmed by western blotting (Fig. 6A). Transwell assays showed that knockdown of GDF-15 enhanced the migration of HTR-8 cells, while overexpression of GDF-15 inhibited migration of HTR-8/SVneo cells (Fig. 6B–D). Further, HTR-8/SVneo cells were treated with 5 ng/mL and 25 ng/mL of recombinant human GDF-15 protein; then, transwell cell migration and wound healing assay were conducted. The results showed that the migration ability of HTR-8/SVneo cells was inhibited after treatment with recombinant human GDF-15 protein (Fig. 6E–H). These results suggest that FXR1 regulates the migration and invasion of trophoblasts via the GDF-15.

## Discussion

Many studies have found that insufficient migration and invasion of trophoblasts are associated with recurrent miscarriage [6–8]. In this study, we explored the function of FXR1 in trophoblast migration during the early stage of pregnancy. The FXR1 protein, found mostly in the cytoplasm, has RNA-binding properties and contains a nucleolar targeting signal. FXR proteins are known to play an important role in post-transcriptional regulation [4, 9, 10, 24, 25] and are associated with the regulation of specific mRNAs in cells and in the pathogenesis of various diseases. Depletion of FXR1 decreased levels of *CCL2* and *IL1b* mRNA and also decreased the ability of THP1 human monocytic leukemic cells to induce cell migration of neighboring monocytic cells [26]. In head and neck squamous cell carcinoma, significant copy number amplification and mRNA overexpression of FXR1 regulates p21 and TERC RNA to bypass p53-mediated cellular senescence [27]. Qian et al. demonstrated that FXR1 executes its regulatory function by forming a novel complex with two other oncogenes, protein kinase C and iota and epithelial cell transforming 2, located in the same amplicon, via distinct binding mechanisms. FXR1 overexpression is regarded as a candidate biomarker predictive of poor survival in multiple solid tumors including NSCLCs and 18 types of human cancers, such as lung, cervix, head, breast, ovarian, and neck squamous carcinoma [28]. Edwin et al. generated an FXR1 KO mouse model. Homozygous FXR1 knockout neonates died shortly after birth, most likely due to cardiac or respiratory failure. Heterozygote FXR1 knockout neonates showed a striated muscle phenotype, displayed a strongly reduced limb musculature, and had a reduced life span of 18 weeks. This suggests that FXR1 may play a role in muscle mRNA transport/translation control, similar to that seen for FMRP in neuronal cells [29]. In our previous study, we explored that FXR1 mRNA expression levels were significantly decreased in trophoblasts from recurrent miscarriage patients and that FXR1 may be involved in the regulation of maternal fetal interface by binding to the 3′-UTR region of COX-2 to represses its expression [12]. In this study, we further confirmed that the level of FXR1 protein expression was downregulated in chorionic villous tissue from the RSA group compared with that in healthy controls. We also explored if FXR1 could regulate trophoblast migration in the early stages of human placental development. The migration ability of HTR-8/SVneo cells and trophoblast outgrowth using an ex vivo extravillous explant culture model was obviously decreased after the knockdown of FXR1.

GDF-15 is associated with cardiovascular disease, inflammation, body weight regulation, and cancer. Serum GDF-15 increased with gestation age, reaching the highest level in the third trimester [15, 16, 30]. The placenta is the only tissue that expresses large amounts of GDF-15 under normal physiological conditions and is produced by trophoblasts, stromal cells, and luminal and glandular epithelial cells of the first trimester decidua [15–17]. Intense GDF-15 immunostaining can be observed in the syncytiotrophoblast of the early pregnant placenta and in the trophoblast cell columns which give rise to the invasive extravillous trophoblast cell lineage [16]. In our study, consistent with previously reported literature, immunohistochemical analysis showed a positive signal for GDF-15 in CTBs in the HCs. Strong expression of GDF-15 was observed in chorionic villous tissue in the RSA group, with the staining mainly distributed in CTBs and STBs. Western blotting showed that the level of the GDF-15 protein was upregulated in chorionic villous tissues from RSA patients compared to that in chorionic villous tissues from the HCs.

The function of GDF-15 in the maternal fetal interface has been reported. GDF-15 was suggested to act to maintain pregnancy by suppressing the production of proinflammatory cytokines [15, 16]. In human trophoblast cells, GDF-15 led to apoptosis and inhibited proliferation [19, 31]. In vitro studies using EVT cells indicated that GDF-15 had overall inhibitory actions on trophoblast invasion through growth inhibition and stimulation of apoptosis [19]. GDF-15 is considered a potent regulator of matrix metalloproteinases, which control the degradation effect of the decidual matrix and thus impact the invasion of trophoblast cells [32]. These data suggest the function of GDF-15 on trophoblasts is to inhibit invasion of the extravillous cytotrophoblast and inhibit villous cytotrophoblast proliferation and differentiation into syncytium at lower concentrations [15, 16, 31, 32]. At high concentrations, trophoblast cells proceed to apoptosis [19, 31]. In our study, we confirmed that knockdown of GDF-15 using siRNA enhanced the migration ability of HTR-8/SVneo cells, while overexpression of GDF-15 inhibited HTR-8/SVneo cell migration. The migration ability of HTR-8 cells was also inhibited after treatment with various lower concentrations of recombinant human GDF-15 protein.

Tong et al*.* found that GDF-15 could predict miscarriage, because serum GDF-15 concentrations were low in the weeks before miscarriage [33]. This is in contrast to what we observed in villi from RSA patients. We found the level of GDF-15 protein was upregulated in chorionic villous tissue from RSA patients compared to the HCs. This contradictory phenomenon can also be found in preeclampsia [16, 30, 34], which is associated with abnormal invasion of trophoblasts and incomplete remodeling of placenta-supplying maternal uterine spiral arteries. There is agreement on the relationship between preeclampsia or miscarriage and maternal serum GDF-15 concentration. We speculate that the placenta is the most likely source of GDF-15, but decidual secretion may also contribute to circulating GDF-15 [16].

In conclusion, we demonstrated here, that the level of FXR1 protein expression was downregulated and GDF-15 expression was upregulated in chorionic villous tissue from the RSA group compared with that from HC group. Using RNA-binding protein immunoprecipitation, we proved that FXR1 may regulate the expression of GDF-15 at the posttranscriptional level by directly binding to the 3′-UTR of GDF-15. Together, our data provide new insights into the function of FXR1 in human placenta trophoblast migration via regulation of GDF-15 expression in trophoblasts and suggest a possible pathological mechanism involved in RSA.

## References

[1] Cakmak H, Taylor HS (2011). Implantation failure: molecular mechanisms and clinical treatment. Hum Reprod Update..

[2] Strickland S, Richards WG (1992). Invasion of the trophoblasts. Cell..

[3] Red-Horse K, Zhou Y, Genbacev O, Prakobphol A, Foulk R, McMaster M, Fisher SJ (2004). Trophoblast differentiation during embryo implantation and formation of the maternal-fetal interface. J Clin Invest..

[4] Pijnenborg R, Dixon G, Robertson WB, Brosens I (1980). Trophoblastic invasion of human decidua from 8 to 18 weeks of pregnancy. Placenta..

[5] Katz VL, Kuller JA (1994). Recurrent miscarriage. Am J Perinatol..

[6] Rai R, Regan L (2006). Recurrent miscarriage. Lancet..

[7] Knöfler M, Haider S, Saleh L, Pollheimer J, Gamage TKJB, James J (2019). Human placenta and trophoblast development: key molecular mechanisms and model systems. Cell Mol Life Sci..

[8] Staun-Ram E, Shalev E (2005). Human trophoblast function during the implantation process. Reprod Biol Endocrinol..

[9] Menkhorst E, Winship A, Van Sinderen M, Dimitriadis E (2016). Human extravillous trophoblast invasion: intrinsic and extrinsic regulation. Reprod Fertil Dev..

[10] Siomi MC, Siomi H, Sauer WH, Srinivasan S, Nussbaum RL, Dreyfuss G (1995). FXR1, an autosomal homolog of the fragile X mental retardation gene. EMBO J..

[11] Bardoni B, Schenck A, Mandel JL (2001). The Fragile X mental retardation protein. Brain Res Bull..

[12] Li XC, Song MF, Sun F, Tian FJ, Wang YM, Wang BY, Chen JH (2018). Fragile X-related protein 1 (FXR1) regulates cyclooxygenase-2 (COX-2) expression at the maternal-fetal interface. Reprod Fertil Dev..

[13] Lawton LN, Bonaldo MF, Jelenc PC (1997). Identification of a novel member of the TGF-beta superfamily highly expressed in human placenta. Gene..

[14] Fairlie WD, Moore AG, Bauskin AR, Russell PK, Zhang HP, Breit SN (1999). MIC-1 is a novel TGF-beta superfamily cytokine associated with macrophage activation. J Leukoc Biol..

[15] Moore AG, Brown DA, Fairlie WD, Bauskin AR, Brown PK, Munier ML, Russell PK, Salamonsen LA, Wallace EM, Breit SN (2000). The transforming growth factor-ss superfamily cytokine macrophage inhibitory cytokine-1 is present in high concentrations in the serum of pregnant women. J Clin Endocrinol Metab..

[16] Marjono AB, Brown DA, Horton KE, Wallace EM, Breit SN, Manuelpillai U (2003). Macrophage inhibitory cytokine-1 in gestational tissues and maternal serum in normal and pre-eclamptic pregnancy. Placenta..

[17] Segerer SE, Rieger L, Kapp M, Dombrowski Y, Müller N, Dietl J, Kämmerer U (2012). MIC-1 (a multifunctional modulator of dendritic cell phenotype and function) is produced by decidual stromal cells and trophoblasts. Hum Reprod..

[18] Jones RL, Stoikos C, Findlay JK, Salamonsen LA (2006). TGF-beta superfamily expression and actions in the endometrium and placenta. Reproduction..

[19] Morrish DW, Dakour J, Li H (2001). Life and death in the placenta: new peptides and genes regulating human syncytiotrophoblast and extravillous cytotrophoblast lineage formation and renewal. Curr Protein Pept Sci..

[20] Graham CH, Hawley TS, Hawley RG (1993). Establishment and characterization of first trimester human trophoblast cells with extended lifespan. Exp Cell Res..

[21] Li W, Liu D, Chang W (2014). Role of IGF2BP3 in trophoblast cell invasion and migration. Cell Death Dis.

[22] Zhang Y, Jin F, Li XC, Shen FJ, Ma XL, Wu F, Zhang SM, Zeng WH, Liu XR, Fan JX, Lin Y, Tian FJ (2017). The YY1-HOTAIR-MMP2 signaling axis controls trophoblast invasion at the maternal-fetal interface. Mol Ther..

[23] Li XC, Jin F, Wang BY, Yin XJ, Hong W, Tian FJ (2019). The m6A demethylase ALKBH5 controls trophoblast invasion at the maternal-fetal interface by regulating the stability of CYR61 mRNA. Theranostics..

[24] Zhang Y, O’Connor JP, Siomi MC (1995). The fragile X mental retardation syndrome protein interacts with novel homologs FXR1 and FXR2. EMBO J..

[25] Garnon J, Lachance C, Di Marco S (2005). Fragile X-related protein FXR1P regulates proinflammatory cytokine tumor necrosis factor expression at the post-transcriptional level. J Biol Chem..

[26] Le Tonqueze O, Kollu S, Lee S, Al-Salah M, Truesdell SS, Vasudevan S (2016). Regulation of monocyte induced cell migration by the RNA binding protein, FXR1. Cell Cycle..

[27] Majumder M, House R, Palanisamy N, et al. RNA-binding protein FXR1 regulates p21 and TERC RNA to bypass p53-mediated cellular senescence in OSCC [published correction appears in PLoS Genet. 2016;12 (10) 10.1371/journal.pgen.1006411 PMC5081202 27783624

[28] Qian J, Hassanein M, Hoeksema MD, Harris BK, Zou Y, Chen H, Lu P, Eisenberg R, Wang J, Espinosa A, Ji X, Harris FT, Rahman SMJ, Massion PP (2015). The RNA binding protein FXR1 is a new driver in the 3q26-29 amplicon and predicts poor prognosis in human cancers. Proc Natl Acad Sci U S A..

[29] Mientjes EJ, Willemsen R, Kirkpatrick LL, Nieuwenhuizen IM, Hoogeveen-Westerveld M, Verweij M, Reis S, Bardoni B, Hoogeveen AT, Oostra BA, Nelson DL (2004). Fxr1 knockout mice show a striated muscle phenotype: implications for Fxr1p function in vivo. Hum Mol Genet..

[30] Chen Q, Wang Y, Zhao M, Hyett J, da Silva Costa F, Nie G (2016). Serum levels of GDF-15 are reduced in preeclampsia and the reduction is more profound in late-onset than early-onset cases. Cytokine..

[31] Li S, Wang Y, Cao B, Wu Y, Ji L, Li YX, Liu M, Zhao Y, Qiao J, Wang H, Wang H, Han C, Wang YL (2014). Maturation of growth differentiation factor 15 in human placental trophoblast cells depends on the interaction with matrix metalloproteinase-26. J Clin Endocrinol Metab..

[32] Knöfler M (2010). Critical growth factors and signalling pathways controlling human trophoblast invasion. Int J Dev Biol..

[33] Tong S, Marjono B, Brown DA, Mulvey S, Breit SN, Manuelpillai U, Wallace EM (2004). Serum concentrations of macrophage inhibitory cytokine 1 (MIC 1) as a predictor of miscarriage. Lancet..

[34] Sugulle M, Dechend R, Herse F, Weedon-Fekjaer MS, Johnsen GM, Brosnihan KB, Anton L, Luft FC, Wollert KC, Kempf T, Staff AC (2009). Circulating and placental growth-differentiation factor 15 in preeclampsia and in pregnancy complicated by diabetes mellitus. Hypertension..

